# Ophthalmological Impairments at Five and a Half Years after Preterm Birth: EPIPAGE-2 Cohort Study

**DOI:** 10.3390/jcm11082139

**Published:** 2022-04-11

**Authors:** Thibaut Chapron, Véronique Pierrat, Georges Caputo, Mathilde Letouzey, Elsa Kermorvant-Duchemin, Youssef Abdelmassih, William Beaumont, Amandine Barjol, Guylene Le Meur, Valérie Benhamou, Laetitia Marchand-Martin, Pierre-Yves Ancel, Héloïse Torchin

**Affiliations:** 1 CRESS, Obstetrical Perinatal and Pediatric Epidemiology Research Team, EPOPé, INSERM, INRAE, Université Paris Cité, 75004 Paris, France; veronique.pierrat@inserm.fr (V.P.); mathilde.letouzey@inserm.fr (M.L.); valerie.benhamou@inserm.fr (V.B.); laetitia.marchand@inserm.fr (L.M.-M.); pierre-yves.ancel@inserm.fr (P.-Y.A.); heloise.torchin@inserm.fr (H.T.); 2 Pediatric Ophthalmology Department, Rothschild Foundation Hospital, 25 rue Manin, CEDEX 19, 75940 Paris, France; gcaputo@for.paris (G.C.); yabdelmassih@for.paris (Y.A.); wbeaumont@for.paris (W.B.); abarjol@for.paris (A.B.); 3 CHU Lille, Department of Neonatal Medicine, Jeanne de Flandre Hospital, 59000 Lille, France; 4 Department of Neonatal Medicine, Poissy Saint Germain Hospital, 78300 Poissy, France; 5 Department of Neonatal Medicine, Necker–Enfants Malades University Hospital, AP-HP, Université Paris Cité, 75015 Paris, France; elsa.kermorvant@aphp.fr; 6 Clinique Ophtalmologique, CHU Nantes, 1 Place Alexis Ricordeau, CEDEX 1, 44093 Nantes, France; guylene.lemeur@chu-nantes.fr; 7 Clinical Research Unit, Center for Clinical Investigation P1419, APHP, CUP, 75014 Paris, France; 8 Department of Neonatal Medicine, Cochin-Port Royal Hospital, FHU PREMA, AP-HP Centre, 75014 Paris, France

**Keywords:** vision, retina, strabismus, refractive errors, visual acuity, cohort study, child, preterm

## Abstract

We report the 5^1/2^ year prevalence of visual and oculomotor impairments in preterm children born at 24–34 weeks’ gestation (WG) using the population-based cohort study EPIPAGE-2, set in France, 2011. The main outcomes were imputed prevalence of refractive errors (REs), strabismus, and binocular visual acuity (VA). Children were clinically assessed by specially trained pediatricians. The population was also analyzed in terms of cerebral palsy at 5^1/2^ years (no CP, stage 1, stage 2, or stage 3–5) and retinopathy of prematurity in the neonatal period (no ROP, stage 1 or 2, or severe ROP). Among the 4441 children included, 2718 (weighted percentage 58.7%) were clinically assessed. REs were reported in 43.1% (95% confidence interval 37.6–48.4), 35.2% (32.7–37.6), and 28.4% (25.0–31.8) of children born at 24–26, 27–31, and 32–34 WG (*p* < 0.01), respectively; strabismus rates were 19.5% (14.6–24.4), 14.8% (12.9–16.7), and 8.3% (6.2–10.4) (*p* < 0.001), respectively. Moderate/severe visual deficiencies (VA < 3.2/10) were present in 1.7% (0.2–3.3) of children born at 24–26 WG, and in less than 1% in other groups. A suboptimal VA 5/10–6.3/10 was measured in 40.6% (35.3–45.8) of children born at 24–26 WG, 35.8% (33.5–38.1) at 27–31 WG, and 33.7% (30.4–37.0) at 32–34 WG. CP and ROP were associated with strabismus and RE. The association between CP and VA was strong, while it was not observed for ROP. In this large cohort of preterm-born children, we found a high prevalence of RE and strabismus regardless of WG, supporting the need for specific attention in this population. High prevalence of suboptimal VA could be challenging for these children at the age of reading and writing acquisition.

## 1. Introduction

Over the last decade, the survival of preterm-born children has improved, but they remain at high risk of neuro-sensorial impairment [1,2,3]. Cognitive impairments and cerebral palsy (CP) have been widely studied, whereas sensorial impairments—and especially ophthalmological impairments—have not been equally reported. The association between strabismus and CP is well known [4]. Recent reports regarding long-term ophthalmological impairments have focused either on extremely preterm-born children or on children with retinopathy of prematurity (ROP) [5,6,7,8]. For instance, the Swedish EXPRESS cohort study found that 17% of children born before 27 weeks’ gestation (WG) developed strabismus, and 30% had refractive errors at 6.5 years of age [6]. Moreover, studies on limited numbers of children have shown that prematurity per se increases the risk of strabismus, refractive errors, and visual function impairment, including decreases in visual acuity, contrast sensitivity, visual field, and color vision [9,10,11]. These ophthalmological impairments can further threaten the early acquisitions of these children, who are at high risk of cognitive disabilities that could be associated with visuoperceptual disorders [12,13]. However, recent epidemiological evidence supporting screening guidelines for visual impairments in preterm-born children is lacking. In many countries, universal ophthalmological screening performed by trained health professionals is recommended before the age of 5 [14,15]. In France, additional screening by ophthalmologists is recommended for preterm-born children at the ages of 1, 3, and 5 years, regardless of gestational age (GA) at birth [16]. In comparison, examination by ophthalmologists is not specifically required for preterm-born children in Great Britain, but special attention to children with severe intracranial hemorrhage, neonatal sepsis, or ROP is recommended [17].

In this paper, we report the prevalence of visual and oculomotor impairments at 5^½^ years of age in children born in 2011 between 24 and 34 WG and included in the EPIPAGE-2 cohort study.

## 2. Materials and Methods

### 2.1. Study Design and Population

Data were extracted from EPIPAGE-2—a French prospective population-based cohort study. All children born at 22 to 34 WG in 2011 were eligible for inclusion [18,19]. Inclusion periods differed depending on GA. The initial participation rate was 93%. Survivors were followed at 2 [20] and 5^1/2^ years [1].

To obtain reference data at 5^½^ years, a sample of 592 term-born children (born after 36 WG) from the ELFE (*Étude Longitudinale Française depuis l’Enfance*) cohort—a French cohort of more than 18,000 children born in 2011 [21]—were assessed following the same protocol [1].

### 2.2. Data Collection at 5^½^ Years of Age

All included children underwent a clinical examination by a pediatrician in 110 centers specifically opened for the study, and their parents were interviewed. The pediatricians were trained to ensure homogeneity in their evaluation, but were not blinded to the children’s GA. If the child was unable to participate due to a severe disability, parental permission was sought to contact rehabilitation centers to provide information enabling classification of disabilities. If parents refused medical assessment, or if the assessment team was not available, parents were asked to complete a standardized questionnaire.

### 2.3. Visual Outcomes at 5^½^ Years of Age

Data concerning ophthalmological impairments were collected through interviews with parents and physical examination performed by specially trained pediatricians. When available, clinical examination was completed with ophthalmological reports extracted from the child’s health records. Four categories of ophthalmological impairments were collected using this collection method: refractive errors, strabismus, amblyopia, and nystagmus. Refractive errors were defined as a prescription of glasses by an ophthalmologist. Refractive errors were classified as hyperopia without astigmatism, hyperopia with astigmatism, myopia with or without astigmatism, or isolated astigmatism. Information on refractive error was extracted from ophthalmological reports or glasses prescription. All types of strabismus were included, and amblyopia was defined as a patching therapy in progress.

During physical examinations performed by the pediatricians, visual acuity was measured using the Sander–Zanlonghi scale (see Snellen equivalent and logMAR conversion in Table S1) [22]. If children wore glasses, corrected visual acuity was measured; otherwise, uncorrected visual acuity was reported. Both monocular and binocular visual acuity were measured. Only binocular visual acuity is reported in this paper. Visual deficiency was classified according to the World Health Organization (WHO) criteria: severe/moderate visual deficiency for binocular visual acuity measured under 3.2/10, and mild for binocular visual acuity measured between 3.2/10 and 4/10 [23]. In children without visual deficiency according to the WHO, measured binocular visual acuity was divided into three groups: 5/10–6.3/10, 8/10, and 10/10. Visual acuity of 5/10–8/10 corresponds to suboptimal visual acuity [24]. The composite outcome “at least one ophthalmological impairment” included children with either refractive errors, strabismus, amblyopia or nystagmus, or mild-to-severe visual deficiency.

### 2.4. Maternal and Children Characteristics

The individual characteristics used in this study were as follows: maternal variables (parents’ socio-professional category, mother’s educational level, mother’s working situation); neonatal characteristics (GA; small-for-GA (SGA), defined as a birthweight below the 10th percentile [25]); the presence of severe neonatal morbidities, including severe cerebral lesions, severe bronchopulmonary dysplasia, or severe necrotizing enterocolitis [26]; and the presence of ROP with its worst staging according to the international classification [27]. CP was assessed during the medical evaluation, reported according to the diagnostic criteria of the Surveillance of CP in Europe [28], and graded using the Gross Motor Function Classification System (GMFCS) [29]. Subgroups were categorized as no CP, stage 1, stage 2, or stage 3–5 CP. ROP screening was performed for children under 32 WG, and reported in this study as no ROP, stage 1 or 2 ROP, or severe ROP [30].

### 2.5. Data Management and Statistics

Outcomes among children assessed at 5^½^ years old are presented according to groups of inclusion at birth, based on GA (24^+0^–26^+6^, 27^+0^–31^+6^, and 32^+0^–34^+6^ WG). Characteristics of children evaluated at 5^1/2^ years and of those lost to follow-up were compared. Percentages, means, and odds ratios (ORs, with 95% confidence intervals (CIs)) were weighted by recruitment period. All tests were two-sided; *p*-values were given for comparison between the three groups of preterm children; a *p*-value < 0.05 was considered statistically significant. Statistical analyses were performed using SAS v9.4 software.

We report the prevalence of ophthalmological impairments and visual deficiency. Groups of preterm-born children were compared using the chi-squared test for categorical variables. Percentages from the ELFE cohort were weighted to account for the sampling method of the cohort and socioeconomic characteristics [1]. Imputation models included variables potentially associated with loss to follow-up or with visual outcomes, based on neonatal characteristics, 2-year outcomes data, and data from the 5^1/2^ years parental questionnaire (Table S2). These outcomes were examined by pediatricians or psychologists. Visual acuity measurements were converted to logMAR units for the multiple imputation process. Missing data were imputed by chained equations using the SAS multiple imputation procedure [31]. We generated 50 independent imputed datasets with 30 iterations each. Estimates were pooled according to Rubin’s rule [32].

We then describe ophthalmological impairments in subgroups of children according to CP and ROP status. Associations between ophthalmological impairments and CP or ROP were studied using GEE logistic regression models to account for the non-independence of outcomes related to multiple births. Models were adjusted according to GA and the presence of at least one severe neonatal morbidity.

### 2.6. Ethics

All parents signed informed consent to participate in the study. This study was approved by the National Data Protection Authority (Commission Nationale de l’Informatique et des Libertés, DR-2016-290) and by ethics committees (CCTIRS 10. 623, CPP Ile-de-France 2016-A00333-48).

## 3. Results

### 3.1. Population

**Figure 1:**
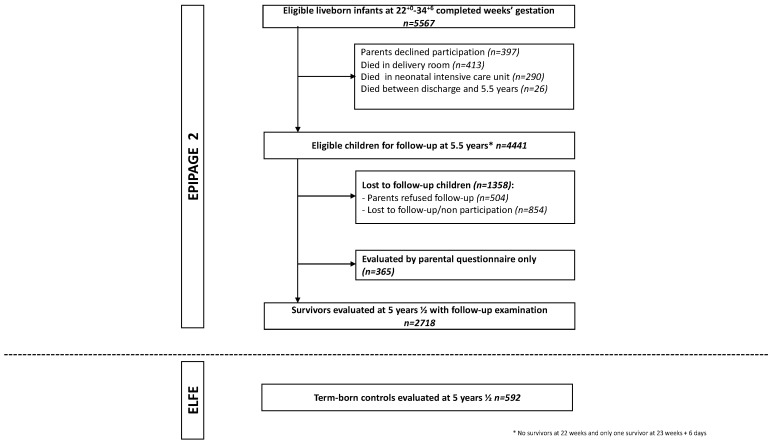
Study population.

At 5^½^ years of age, 4441 children were alive, of whom 2718 (weighted percentage 58.7%) attended the follow-up examination (Figure 1). Children lost to follow-up were predominantly girls, singletons, and born at 32–34 WG; their parents were younger, born outside France, and had lower levels of education and socioeconomic status. Neonatal morbidities and ophthalmological impairments at 2 years were similar between both groups, except for a higher rate of strabismus in the group lost to follow-up (Table S3).

### 3.2. Ophthalmological Impairments and Binocular Visual Acuity

After imputation, the prevalence of at least one ophthalmological impairment was 48.5%, 39.7%, and 32.6% of children born at 24–26, 27–31, and 32–34 WG, respectively (*p* < 0.001), compared to 23.8% in term-born children (Table 1).

Refractive error was the most frequent impairment, reported in 43.1%, 35.2%, and 28.4% of children born at 24–26, 27–31, and 32–34 WG, respectively. Among refractive errors, hyperopia was the most frequent refractive error diagnosed in all GA groups (Table S4). In children born at 24–26 WG with complete data, the prevalence of myopia was 7.5%, while that of hyperopia was 14.0%. The imputed prevalence of strabismus was 19.5%, 14.8%, and 8.3% while that of amblyopia was 9.7%, 8.2%, and 6.4%, in children born at 24–26, 27–31, and 32–34 WG, respectively. Among children who underwent visual acuity testing, 783 (weighted percentage 27.6%) had glasses prescribed. Among them, 103 (weighted percentage 3.8%) did not wear them during the exam. Results for this specific group did not differ from those for the other groups (Table S5). After imputation, 10/10 binocular visual acuity was reported in 28.6%, 35.1%, and 36.0% of children born at 24–26, 27–31, and 32–34 WG, respectively, and in 59.7% of term-born children. Severe/moderate visual deficiency rates were 1.7% or less in all groups of preterm-born children. Mild visual deficiency was found in 8.1%, 5.9%, and 5.4%, while suboptimal binocular visual acuity of 5–6.3/10 was found in 40.6%, 35.8%, and 32.3%, of children born at 24–26, 27–31, and 32–34 WG, respectively (Table 1). Tests for trend by GA were not statistically significant in any visual acuity group. The group of children with a prescription for glasses who were wearing them during the exam seemed not to have better visual acuity than the group of children with no prescription for glasses. (Table S5).

Before multiple imputation, the prevalence of strabismus and amblyopia was slightly lower. Other outcomes and visual acuity were similar before and after multiple imputation (Table 1).

### 3.3. Ophthalmological Impairments among Children with CP or Previous History of ROP

Cerebral palsy was diagnosed in 134 (weighted percentage 3.6%) children born preterm, whereas 196 (weighted percentage 3.1%) developed ROP (any stage) in the neonatal period. The prevalence of ophthalmological impairments increased with the presence of CP or ROP, as well as with their severity (Table 2).

The presence of CP was strongly associated with visual deficiency and suboptimal visual acuity, whereas ROP during the neonatal period was not (Table 3).

## 4. Discussion

In this French national cohort of preterm children born in 2011, high rates of ophthalmological impairments were observed at the 5.5-year follow-up. Refractive errors were the most frequent impairments, followed by strabismus, for which the prevalence remained high even in children born at 32–34 WG (8.3%). The prevalence of refractive error and strabismus increased as GA decreased, whereas rates of visual deficiencies were not statistically different between the different GA groups. Mild-to-severe visual deficiency was observed in 5% to 10% of children, but rates of suboptimal visual acuity were high. Rates of 10/10 visual acuity ranged from 28.6% in children born at 24–26 WG to 36.0% in children born at 32–34 WG, compared to 59.7% in term-born children.

Strengths of the EPIPAGE-2 study include the population-based national cohort design with prospective enrolment of all preterm-born babies. Term-born children were also enrolled at 5^½^ years as a reference group. Nearly 60% of preterm-born children alive at 5^½^ years received a medical examination. Multiple imputation was performed based on neonatal characteristics and 2-year outcome data in order to mitigate attrition bias and deliver reliable prevalence estimates for the whole population, including the most disabled infants. Moreover, we studied a broad range of visual outcomes, and not just the most severe. The main limitation of this study is that the ophthalmological assessment was not performed by ophthalmologists. Thus, precise data on the type of strabismus, the degree of refractive error, or more specific visual evaluations (color vision deficiency, contrast sensitivity, visual field, etc.) or visual processing were not available. In the same way, refractive error type descriptions were not the most accurate data. However, we assumed that the prevalence estimates of ophthalmological impairments were correct, since at 5^½^ years of age most children should have received a least a preschool ophthalmological screening, a measurement of visual acuity, and evaluation for strabismus. Moreover, in this study, data were derived from physical examinations performed by pediatricians trained to conduct routine ophthalmological screenings, and completed with information from the children’s health records. This process is therefore similar to real-life screening practices. Prevalence may have been slightly overestimated, but this reflects situations that should be addressed via specialized ophthalmic examination.

Comparisons with previously published studies are difficult, as GA and age of assessment are highly heterogeneous. However, rates of ophthalmological impairments are in the same range. The Swedish EXPRESS cohort, which included only children born before 27 WG, found strabismus in 17% of children at 6^1/2^ years of age, refractive errors in 30%, and severe/moderate visual deficiency in 5% [6]. The 2-year follow-up of children born before 28 WG in the English ELGAN study found a prevalence of strabismus of 14%, but the type and prevalence of strabismus differed depending on age at evaluation [7]. In the Millennium cohort study, the prevalence of strabismus was of 2.1% for all children aged 3 years, and 5.4% for those born before 34 WG, with few children under 32 WG [5].

More than 30% of preterm-born children had suboptimal visual acuity of 5–6.3/10, with no significant association with GA. One explanation for this could be the lack of refractive error screening, but the group of children wearing glasses during the visual acuity exam seemed to have reduced visual acuity compared to the group of children with no glasses prescription. We therefore did not retain this hypothesis.

This reduced visual acuity may be the consequence of impaired foveal development, resulting in the reduction in the size of the avascular zone [33,34,35]. Another explanation could also be the slower and abnormal maturation of visual pathways in this population [36]. Therefore, the visual acuity of preterm-born children may increase over time. This could explain the higher rates of suboptimal visual acuity reported in our study compared to studies that evaluated older children [6,11]. Nevertheless, suboptimal corrected visual acuity could affect reading and writing acquisition in preterm-born children, who also have a higher risk of cognitive disability as well as attentional and dysexecutive disorders [1]. These findings suggest that specific attention be paid to these children, as recommended by the “European standards of care for newborn health” [37]. A more specific ophthalmological examination including structural imaging and prolonged follow-up could help us to better understand these mechanisms.

As expected, children with CP or ROP had higher rates of ophthalmological impairments, even when the conditions were mild. On the one hand, CP was strongly associated with lower visual acuity, which might be explained by the white matter lesions observed on MRI imaging [38]. On the other hand, ROP was associated with strabismus and refractive errors, but not with decreased visual acuity. This may reflect the role of impaired brain development in oculomotor and neuro-visual disorders, as well as improvements in ROP screening and treatment. Ophthalmological screening can be challenging in children with severe cognitive impairment or specific oculomotor dysfunctions due to neurological injury. Thus, follow-up by health care professionals experienced in the care of these children is important.

## 5. Conclusions

In this large cohort, we reported a high prevalence of refractive error and strabismus regardless of GA. One off-note finding is the frequent proportion of preterm-born children presenting with a moderate decrease in corrected visual acuity. These results may help to update guidelines to follow the visual development of children born preterm. While data regarding macula and brain structure development [32,35] may be consistent with these findings, further studies are needed to better understand their mechanisms and their consequences for children’s learning capabilities.

## Figures and Tables

**Table 1:** Ophthalmological impairments and binocular visual acuity measured at 5^1/2^ years by gestational age group among survivors in the EPIPAGE-2 study. Values are percentages (95% confidence interval). Observed and imputed data.

N (Total = 2718) *	24–26 WG †		27–31 WG		32–34 WG		*p* **	Reference Sample Term–Born Children ‡
	CC	MI	CC	MI	CC	MI		CC
	n/N % (95% CI)	% (95% CI)	n/N % (95% CI)	% (95% CI)	n/N % (95% CI)	% (95% CI)		% (95% CI)
At least one ophthalmological impairment §	150/325 46.1 (40.6–51.7)	– 48.5 (43.4–53.6)	621/1666 37.3 (35.0–39.6)	– 39.7 (37.8–41.4)	203/664 30.6 (27.1–34.2)	– 32.6 (30.4–34.2)	<0.001	23.8 (19.1–29.1)
Refractive errors	133/294 45.2 (39.5–51.1)	– 43.1 (37.6–48.4)	560/1514 37.0 (34.5–39.5)	– 35.2 (32.7–37.6)	187/602 31.1 (27.4–35.0)	– 28.4 (25.0–31.8)	<0.001	24.1 (19.1–29.7)
Strabismus	48/291 16.5 (12.4–21.3)	– 19.5 (14.6–24.4)	185/1519 12.2 (10.6–13.9)	– 14.8 (12.9–16.7)	41/609 6.7 (4.9–9.0)	– 8.3 (6.2–10.4)	<0.001	2.8 (1.0–5.3)
Amblyopia	15/278 5.4 (3.1–8.7)	– 9.7 (5.7–13.6)	65/1439 4.5 (3.5–5.7)	– 8.2 (6.2–10.1)	24/594 4.0 (2.6–6.0)	– 6.4 (4.2–8.6)	0.69	0.5 (0.3–2.6)
Nystagmus	3/284 1.1 (0.2–3.1)	– 2.7 (0.8–4.7)	8/1501 0.5 (0.2–1.0)	– 1.6 (0.8–2.3)	4/609 0.7 (0.2–1.7)	– 1.4 (0.5–2.3)	0.76	0.5 (0.3–2.6)
**Visual deficiency**							0.43 $	
Severe and moderate VA <3.2/10	3 1.0 (0.2–2.8)	– 1.7 (0.2–3.3)	7 0.4 (0.2–0.9)	– 0.8 (0.3–1.2)	3 0.5 (0.1–1.3)	– 0.6 (0.0–1.1)		0.7 (0.0–4.0)
Mild VA 3.2/10–4/10	19 6.2 (3.8–9.5)	8.1 (5.2–11.1)	82 5.0 (4.0–6.2)	5.9 (4.8–7.0)	34 5.2 (3.6–7.2)	5.4 (3.8–7.1)		3.0 (1.1–6.3)
None								
VA 5/10–6.3/10	124 40.3 (34.7–46.0)	40.6 (35.3–45.8)	574 35.1 (32.8–37.5)	35.8 (33.5–38.1)	210 32.3 (28.7–36.0)	33.7 (30.4–37.0)		17.7 (13.6–22.4)
VA 8/10	65 21.1 (16.7–26.1)	21.0 (16.7–25.2)	369 22.6 (20.6–24.7)	22.4 (20.6–24.2)	157 24.9 (21.7–28.4)	24.3 (21.2–27.3)		18.9 (14.6–23.8)
VA 10/10	97 31.5 (26.3–37.0)	28.6 (24.0–33.3)	603 36.9 (34.5–39.3)	35.1 (32.8–37.4)	238 37.1 (33.4–41.0)	36.0 (32.5–39.5)		59.7 (53.9–65.4)

WG = weeks’ gestation. CC = complete cases. MI = multiple imputation (4441 children) VA = visual acuity. * Observed data, denominators vary according to the number of missing data for each variable. ** *p* value are given for comparison between the three groups of Gestational Ages. † Including only one survivor born at 23 weeks + 6 days in the group lost to follow-up, no survivors at 23 weeks in the group follow-up at 5. § Presence of at least one ophthalmological impairment including refractive errors, strabismus, amblyopia, nystagmus or any visual disability as defined by the World Health Organization (2021). ‡ Percentages from the ELFE cohort were weighted to account for the sampling method and socio-economic characteristics. $ p for trend performed on each category was >0.05.

**Table 2:** Prevalence of refractive errors and strabismus by cerebral palsy and retinopathy of prematurity subgroups at 5^1/2^ years among survivors in the EPIPAGE-2 study. Values are weighted percentages (95% confidence interval) and odds ratios using a generalized estimating equation adjusted for gestational age and severe neonatal morbidities. Observed and imputed data.

	Cerebral Palsy								
	None		GMFCS–1		GMFCS–2		GMFCS–3–5		
	n/N CC % (95% CI) ORa * (95% CI)	MI ‡ % (95% CI) ORa * (95% CI)	n/N CC % (95% CI) ORa * (95% CI)	MI ‡ % (95% CI) ORa * (95% CI)	n/N CC % (95% CI) ORa * (95% CI)	MI ‡ % (95% CI) ORa * (95% CI)	n/N CC % (95% CI) ORa * (95% CI)	MI ‡ % (95% CI) ORa * (95% CI)	*p*
									
Refractive errors	807/2282 32.6 (30.1–35.3) 1	29.6 (27.2–32.0) 1	29/58 48.8 (30.6–67.2) 1.6 (1.0–2.7)	49.4 (31.2–67.1) 2.0 (1.2–3.5)	19/32 60.7 (36.6–81.4) 2.8 (1.3–6.2)	61.2 (37.9–84.5) 3.2 (1.6–7.9)	24/31 82.4 (64.6–93.7) 6.0 (2.5–14.4)	75.8 (48.1–104) 5.5 (1.4–21.7)	<0.001
Strabismus	232/2294 8.1 (6.7–9.6) 1	9.3 (7.7–10.9) 1	13/56 13.0 (5.5–24.7) 2.6 (1.4–4.8)	21.1 (7.3–34.8) 2.8 (1.5–5.2)	12/33 32.7 (14.0–56.5) 4.6 (2.2–9.8)	42.3 (20.1–64.5) 5.7 (2.7–12.1)	17/29 68.4 (47.7–84.8) 11.3 (4.9–26.0)	64.5 (38.7–90.5) 9.7 (3.5–26.7)	<0.001
	**Retinopathy of prematurity**								
	**None**		**ROP stages 1 or 2**		**Severe ROP** †				
	n/N CC % (95% CI) ORa * (95% CI)	MI ‡ % (95% CI) ORa * (95% CI)	n/N CC % (95% CI) ORa * (95% CI)	MI ‡ % (95% CI) ORa * (95% CI)	n/N CC % (95% CI) ORa * (95% CI)	MI ‡ % (95% CI) ORa * (95% CI)			
									
Refractive errors	801/2237 33.2 (30.6–35.8) 1	30.5 (28.0–33.0) 1	62/149 41.3 (33.3–49.7) 1.1 (0.7–1.5)	41.1 (33.8–48.4) 1.2 (0.9–1.6)	17/24 70.5 (48.6–87.2) 3.1 (1.2–7.8)	63.8 (45.8–81.8) 2.5 (1.2–5.5)			<0.001
Strabismus	244/2249 8.6 (7.2–10.1) 1	10.3 (8.5–12.0) 1	23/150 15.5 (10.1–22.3) 1.2 (0.7–1.9)	19.4 (13.8–24.9) 1.3 (0.9–1.9)	7/20 34.8 (15.3–59.1) 3.0 (1.1–8.2)	43.0 (23.6–62.4) 3.4 (1.5–7.7)			<0.001

CC = complete cases. MI = multiple imputation (4441 children). GMFCS: Gross Motor Function Classification System. ROP: Retinopathy of prematurity. * ORs were adjusted on gestational age and severe neonatal morbidity excluding retinopathy of prematurity and studied by using GEE logistic regression models to account for the non-independence of outcomes related to multiple births. Severe neonatal morbidity was defined as severe bronchopulmonary dysplasia or necrotizing enterocolitis stage 2–3 or intraventricular haemorrhage grade III or IV or cystic periventricular leukomalacia (Ancel 2015). ‡ Percentages and ORs are presented after multiple imputation of all eligible to 5^1/2^ follow-up children to account for missing information at 5^1/2^ years. Estimates were pooled according to Rubin’s rule. † Defined as ROP stage 3 or more or ROP treated.

**Table 3:** Binocular visual acuity by cerebral palsy and retinopathy of prematurity sub-groups at 5^1/2^ years among survivors in the EPIPAGE-2 study. Values are weighted percentages (95% confidence interval) and odds ratios using a generalized estimating equation adjusted for gestational age and severe neonatal morbidities. Observed and imputed data.

Cerebral Palsy									
	No Cerebral Palsy		Stage 1		Stage 2		Stages 3 or 4 or 5		
	CC n/N % (95% CI) ORa * (95% CI)	MI % (95% CI) ORa * (95% CI)	CC n/N % (95% CI) ORa * (95% CI)	MI % (95% CI) ORa * (95% CI)	CC n/N % (95% CI) ORa * (95% CI)	MI % (95% CI) ORa * (95% CI)	CC n/N % (95% CI) ORa * (95% CI)	MI % (95% CI) ORa * (95% CI)	*p*
Visual deficiency									<0.001
Severe and moderate VA <3.2/10	11/2481 0.4 (0.1–0.8) 1	– 0.4 (0.1–0.7) 1	0/57 0 0	– 0 0	0/29 0 0	– 0 0	2/22 23.9 (3.0–63.3) 42.9 (5.4–340.0)	– 14.1 (0.0–28.8) 86.1 (13.4–553.4)	
Mild VA 3.2/10–4/10	120/2481 4.9 (3.8–6.2) 1	– 5.2 (4.0–6.4) 1	4/57 3.5 (0.4–12.1) 1.4 (0.5–3.9)	– 6.3 (0.0–14.0) 1.6 (0.6–4.3)	6/29 22.2 (5.8–49.2) 9.0 (2.6–31.2)	– 20.0 (4.5–35.4) 7.9 (2.8–22.7)	4/22 15.4 (3.8–37.1) 10.7 (2.3–49.1)	– 29.3 (11.5–47.1) 30.3 (8.0–115.4)	
None									
5/10–6.3/10	863/2481 33.1 (30.6–35.6) 1	– 34.2 (31.7–36.6) 1	23/57 47.0 (28.8–65.7) 1.0 (0.5–1.9)	– 43.5 (28.0–58.9) 1.1 (0.7–2.0)	12/29 54.4 (29.8–77.5) 2.3 (0.8–6.7)	– 50.2 (29.7–70.7) 2.7 (1.1–6.8)	9/22 33.7 (15.1–57.0) 2.8 (0.8–10.2)	– 41.5 (22.2–60.8) 6.2 (2.0–19.5)	
8/10	578/2481 24.4 (22.2–26.8) 1	– 24.0 (21.8–26.1) 1	8/57 12.0 (3.6–27.1) 0.6 (0.3–1.3)	– 16.4 (5.0–27.9) 0.8 (0.4–1.6)	5/29 10.6 (2.3–27.7) 1.4 (0.4–4.9)	– 14.8 (2.1–27.6) 1.7 (0.6–4.4)	4/22 15.4 (3.8–37.1) 1.9 (0.5–6.8)	– 9.0 (0.0–18.3) 2.3 (0.7–8.2)	
10/10	909/2481 37.2 (34.6–39.8) 1	– 36.2 (33.7–38.7) 1	22/57 37.5 (21.0–56.5) 1	– 32.9 (18.3–47.6) 1	6/29 12.8 (3.4–30.5) 1	– 12.9 (1.2–24.6) 1	3/22 11.6 (2.0–32.3) 1	– 6.1 (0.0–15.2) 1	
**Retinopathy of prematurity**									
	**No ROP**		**ROP stages 1 or 2**		**Severe ROP†**				
	CC n/N % (95% CI) ORa* (95% CI)	MI % (95% CI) ORa* (95% CI)	CC n/N % (95% CI) ORa* (95% CI)	MI % (95% CI) ORa* (95% CI)	CC n/N % (95% CI) ORa* (95% CI)	MI % (95% CI) ORa* (95% CI)			
Visual deficiency									0.11
Severe and moderate VA <3.2/10	13/2425 0.50 (0.2–1.0) 1	– 0.6 (0.2–1.0) 1	0/152 0 0	– 0 0	0/20 0 0	– 0 0			
Mild VA 3.2/10–4/10	121/2425 5.1 (4.0–6.4) 1	– 5.6 (4.3–6.8) 1	12/152 7.7 (4.0–13.1) 1.5 (0.7–3.1)	– 9.2 (5.0–13.4) 1.7 (0.9–3.2)	2/20 10.6 (1.4–32.5) 1.7 (0.3–9.1)	– 15.4 (1.6–29.2) 2.5 (0.7–9.7)			
None									
5/10–6.3/10	842/2425 33.3 (30.8–35.9) 1	– 34.4 (32.0–36.8) 1	59/152 39.3 (31.4–47.5) 1.2 (0.8–1.8)	– 40.6 (33.5–47.8) 1.3 (0.9–1.9)	8/20 39.4 (18.6–63.4) 0.7 (0.3–2.1)	– 41.2 (23.5–59.0) 1.2 (0.5–3.1)			
8/10	559/2425 24.1 (21.8–26.5) 1	– 23.6 (21.4–25.8) 1	35/152 22.8 (16.4–30.3) 1.2 (0.7–1.9)	– 21.6 (15.5–27.6) 1.2 (0.7–1.8)	3/20 15.2 (3.3–38.1) 0.6 (0.2–2.3)	– 16.2 (3.1–29.2) 0.9 (0.3–2.7)			
10/10	890/2425 37.0 (34.4–39.6) 1	– 35.8 (33.3–38.3) 1	46/152 30.3 (23.1–38.3) 1	– 27.7 (21.1–34.2) 1	7/20 34.8 (15.3–59.1) 1	– 24.0 (9.2–38.6) 1			

CC = complete cases. MI = multiple imputation (4441 children). ROP: Retinopathy of prematurity. VA = visual acuity. * OR were adjusted on gestational age and severe neonatal morbidity. Severe neonatal morbidity was defined as severe bronchopulmonary dysplasia or necrotizing enterocolitis stage 2–3 or any of the following severe cerebral abnormalities on cranial ultrasonography: intraventricular haemorrhage grade III or IV or cystic periventricular leukomalacia (Ancel 2015). † Defined as ROP stage 3 or ROP treated.

## Data Availability

The data presented in this study are available upon request from the corresponding author. The data are not publicly available due to privacy restrictions.

## Supplementary Materials

The following supporting information can be downloaded at: https://www.mdpi.com/article/10.3390/jcm11082139/s1: Table S1: Conversion table between Sander-Zanlonghi scale and Snellen scale and logMar equivalent; Table S2: Type of variable, model used to predict missing data, and percentages of values missing for each variable included in the imputation model (N = 4441 survivors at 5 years eligible for follow-up); Table S3: Comparison of participating and nonparticipating children eligible for follow-up at 5.5 years, N = 4441; Table S4: Report of refractive errors type depending on gestational age group at birth in preterm live births survivors at 5.5 years who underwent medical exam; Table S5: Comparison of preterm live birth survivors’ visual acuity at 5.5 years by glasses status.
