# Supplementary material for: Ophthalmological Impairments at Five and a Half Years after Preterm Birth: EPIPAGE-2 Cohort Study
Source: J Clin Med. 2022 Apr 11;11(8):2139. doi: 10.3390/jcm11082139 (PMC9027367; doi:10.3390/jcm11082139)
Supplement: Supplementary file 1 [file jcm-11-02139-s001.zip › Table S1.pdf]

Table S1: Conversion table between Sander-Zanlonghi scale and Snellen scale and logMar equivalent

Decimal Sander-Zanlonghi	Snellen equivalent	logMar conversion
3.2/10	20/60	0.5
4/10	20/50	0.4
5/10	20/40	0.3
6.3/10	20/30	0.2
8/10	20/25	0.1
10/10	20/20	0.0
