# Supplementary material for: Ophthalmological Impairments at Five and a Half Years after Preterm Birth: EPIPAGE-2 Cohort Study
Source: J Clin Med. 2022 Apr 11;11(8):2139. doi: 10.3390/jcm11082139 (PMC9027367; doi:10.3390/jcm11082139)
Supplement: Supplementary file 1 [file jcm-11-02139-s001.zip › Table S2.pdf]

Table S2. Type of variable, model used to predict missing data, and percentages of values missing for each variable included in the imputation model (N=4441 survivors at 5 years eligible for follow-up).

Variable	Type of variable	Model used to predict missing data	Percentages of values missing, among survivors at 5 years
**Perinatal characteristics**			
Birth region	Categorical	No missing data	0%
Gestational age	Continuous	No missing data	0%
Maternal age at birth	Categorical (3 categories)	No missing data	0%
Primiparous	Binary	Logistic regression	1%
Maternal counrty of birth	Categorical (4 categories)	Multinomial regression	1%
Socio-economic status*	Categorical (5 categories)	Multinomial regression	5%
Mother level of education	Categorical (5 categories)	Multinomial regression	7%
Living as a couple	Binary	Logistic regression	5%
Tabacco during pregnancy	Categorical (3 categories)	Multinomial regression	5%
Antenatal steroids	Binary	Logistic regression	2%
Multiple pregnancy	Binary	No missing data	0%
Caesarean section	Binary	Logistic regression	<1%
Sex	Binary	Logistic regression	<1%
Small-for-gestational age†	Binary	Logistic regression	<1%
Major congenital malformation	Binary	No missing data	0%
Inborn	Binary	No missing data	0%
Surfactant	Binary	Logistic regression	1%
Severe cerebral lesions	Binary	Logistic regression	1%
Severe bronchopulmonary dysplasia	Binary	Logistic regression	3%
Severe necrotising enterocolitis	Binary	Logistic regression	2%
Retinopathy of prematurity	Categorical (3 categories)	Multinomial regression	23%
Late onset sepsis	Binary	Logistic regression	1%
Extra Uterine Growth Retardation	Categorical (3 categories)	No missing data	0%
Breastmilk at discharge	Categorical (3 categories)	Multinomial regression	7%
**At 2 years CA**			
Cerebral Palsy	Categorical (5 categories)	Multinomial regression	19%
Hearing disabilities	Categorical (3 categories)	Multinomial regression	21%
Visual disabilities	Categorical (3 categories)	Multinomial regression	23%
ASQ Communication	Continuous	Predictive mean matching	18%
ASQ Gross motor score	Continuous	Predictive mean matching	20%
ASQ Fine motor score	Continuous	Predictive mean matching	21%
ASQ Problem solving score	Continuous	Predictive mean matching	21%
ASQ Personal-social	Continuous	Predictive mean matching	21%
Small lexicon size	Binary	Logistic regression	23%
ASQ alert	Binary	Logistic regression	23%
Medical follow-up by ophthalmologist	Binary	Logistic regression	27%
Strabismus	Binary	Logistic regression	20%
Wear glasses	Binary	Logistic regression	21%
Parents concerns about health	Binary	Logistic regression	21%
**At 5 years**			
Cerebral Palsy	Categorical (5 categories)	Multinomial regression	31%
Hearing disabilities	Categorical (4 categories)	Multinomial regression	32%
Visual disabilities	Categorical (5 categories)	Multinomial regression	34%
Visual acuity right eye	Continuous	Predictive mean matching	42%
Visual acuity left eye	Continuous	Predictive mean matching	44%
Strabismus	Binnary	Logistic regression	46%
Refractive errors	Binnary	Logistic regression	46%
Nystagmus	Binnary	Logistic regression	46%
Amblyopia	Binnary	Logistic regression	48%
M-ABC2 Total score	Continuous	Predictive mean matching	42%
Global SDQ	Continuous	Predictive mean matching	40%
In education	Binary	Logistic regression	32%

Support at school, special schooling	Categorical (3 categories)	Multinomial regression	32%
Specific intervention‡	Binary	Logistic regression	33%
Concerns about anxiety	Binary	Logistic regression	34%
Concerns about coordination	Binary	Logistic regression	34%
Concerns about learning	Binary	Logistic regression	34%
Concerns about social interaction	Binary	Logistic regression	34%
Concerns about langage	Binary	Logistic regression	34%
Concerns about behavioral	Binary	Logistic regression	33%
Ophthalmological exam during last year	Binary	Logistic regression	34%
At least one smoker at home	Binary	Logistic regression	38%

ASQ=Ages and Stages questionnaire; MABC-2=Movement Assessment Battery for Children- Second Edition ; SDQ= Strengths and difficulties questionnaire;
All variables were included as a predictor of all imputation models.

* Defined as the highest occupational status between occupations of the mother and the father, or mother only if living alone.

† Small-for- gestational age was defined as birth weight less than the 10th percentile for gestational age and sex based on French intrauterine growth curves (Ego 2016).

‡ At least two consultation with a psychologist, psychiatrist, orthoptist, speech therapist, occupational therapist, physiotherapist, during the twelve last month or a follow-up at medico-psychologist-center or special schooling.
