# Supplementary material for: Ophthalmological Impairments at Five and a Half Years after Preterm Birth: EPIPAGE-2 Cohort Study
Source: J Clin Med. 2022 Apr 11;11(8):2139. doi: 10.3390/jcm11082139 (PMC9027367; doi:10.3390/jcm11082139)
Supplement: Supplementary file 1 [file jcm-11-02139-s001.zip › Table S3.pdf]

Table S3. Comparison of participating and nonparticipating children eligible for follow-up at 5.5 years, N=4441. Values are number of events/number in group and percentage, observed data*.

	Follow-up at 5.5 years		Lost to follow-up for medical exam		
	n=2718		n=1723		Chi2 p-value
**Gestational age**					
24-26 weeks†	340/2718	4.6	204/1723	4.0	<0.001
27-31 weeks	1706/2718	31.3	1004/1723	26.2	
32-34 weeks	672/2718	64.1	515/1723	69.9	
**Maternal characteristics, at birth**					
**Maternal age**					
<25 years	376/2718	11.6	407/1723	22.0	<0.001
25-34 years	1710/2718	63.7	958/1723	58.6	
≥35 years	632/2718	24.7	358/1723	19.5	
**Mother born in France**	2407/2577	94.5	1411/1624	86.1	<0.001
**Living with a patner**	2725/2925	94.1	1093/1276	84.8	<0.001
**Parents' socio-economic status‡**					
Executive manager	671/2607	26.9	250/1593	16.7	<0.001
Intermediate	644/2607	25.8	239/1593	16.0	
Administrative, public service, self-employed, students	666/2607	25.3	484/1593	30.8	
Shop assistants, service workers	322/2607	11.5	279/1593	17.3	
Manual workers	260/2607	8.8	244/1593	14.2	
Unemployed	44/2607	1.7	97/1593	5.1	
**Mother level of education**					
< High school	713/2636	25.6	631/1476	40.9	<0.001
High school	543/2636	20.5	348/1476	23.7	
A-level +1 or +2	566/2636	21.7	227/1476	15.8	
>= A-level +3	814/2636	32.1	270/1476	19.6	
**Multiple pregnancy**	946/2718	37.6	550/1723	32.8	0.01
**Neonatal factors**					
**Male gender**	943/2718	33.9	573/1723	32.4	0.03
**Small-for-gestational age§**	1775/2718	66.1	1149/1723	67.6	0.43
**Severe neonatal morbidities¶**	337/2600	7.1	196/1527	5.9	0.13
**2 years**					
**Cerebral palsy**	89/2547	2.3	48/1051	2.3	0.93
**Strabismus**	141/1515	4.9	89/1022	7.0	0.05
**Refraction error**	187/2493	6.1	87/1019	7.7	0.20
**Examination by an ophthalmologist**	258/2305	9.2	120/923	11.1	0.20
**Ages and Stages Questionnaire below threshold****	961/2424	36.3	400/983	35.1	0.60

ASQ= Ages and Stages Questionnaire.
* Observed data, denominators vary according to the number of missing data for each variable. Percentages are weighted to took into account the differences in survey design between gestational age groups.
† Including only one survivor born at 23 weeks + 6 days in the group lost to follow-up, no survivors at 23 weeks in the group follow-up at 5.
‡ Defined as the highest occupational status between occupations of the mother and the father, or mother only if living alone.
§ Small-for- gestational age was defined as birth weight less than the 10th percentile for gestational age and sex based on French intrauterine growth curves (Ego 2016).
¶ Severe neonatal morbidity was defined as severe bronchopulmonary dysplasia or necrotizing enterocolitis stage 2-3 or severe retinopathy of prematurity stage >3 or any of the following severe cerebral abnormalities on cranial ultrasonography: intraventricular haemorrhage grade III or IV or cystic periventricular leukomalacia (Ancel 2015).
** Below threshold of the US ASQ-3 reference.
