# Supplementary material for: Ophthalmological Impairments at Five and a Half Years after Preterm Birth: EPIPAGE-2 Cohort Study
Source: J Clin Med. 2022 Apr 11;11(8):2139. doi: 10.3390/jcm11082139 (PMC9027367; doi:10.3390/jcm11082139)
Supplement: Supplementary file 1 [file jcm-11-02139-s001.zip › Table S4.pdf]

Table S4. Report of refractive errors type depending on gestational age group at birth in preterm live births survivors at 5.5 years who underwent medical exam. Values are number of events/number in group and percentage, observed data*.

		24-26 weeks	27-31 weeks	32-34 weeks		Reference sample born at term
		n/N<br>% (95% CI)	n/N<br>% (95% CI)	n/N<br>% (95% CI)	p	n/N<br>% (95% CI)
Refraction error type	Isolated Hyperopia	39/279<br>14.0<br>(10.1 to 18.6)	231/1460<br>15.8<br>(14.0 to 17.8)	56/576<br>9.7<br>(7.4 to 12.4)	<.001	57/510<br>8.8<br>(6.1-12.1)
	Myopia and myopia with astigmatism	24/279<br>7.5<br>(4.7 to 11.3)	45/1460<br>3.1<br>(2.3 to 4.1)	19/576<br>3.3<br>(2.0 to 5.1)		10/510<br>3.0<br>(1.0 to 6.8)
	Isolated Astigmatism	14/279<br>5.0<br>(2.8 to 8.3)	75/1460<br>5.1<br>(4.1 to 6.4)	28/576<br>4.9<br>(3.3 to 7.0)		19/510<br>3.0<br>(1.6 to 4.9)
	Hyperopia with astigmatism	21/279<br>7.5<br>(4.7 to 11.3)	94/1460<br>6.4<br>(5.2 to 7.8)	35/576<br>6.1<br>(4.3 to 8.4)		28/510<br>5.5<br>(3.1-8.8)

* Observed data, denominators vary according to the number of missing data for each variable.
