# Supplementary material for: Ophthalmological Impairments at Five and a Half Years after Preterm Birth: EPIPAGE-2 Cohort Study
Source: J Clin Med. 2022 Apr 11;11(8):2139. doi: 10.3390/jcm11082139 (PMC9027367; doi:10.3390/jcm11082139)
Supplement: Supplementary file 1 [file jcm-11-02139-s001.zip › Table S5.pdf]

Table S5. Comparison of preterm live birth survivors' visual acuity at 5.5 years by glasses status. Values are the number of events/number in group and percentage; observed data*.

(Ntotal=2570)	Wearing glasses N=677			No prescription of glasses N=1791			Glasses prescribed but not worn during exam N=102		
Binocular visual acuity	n/N	%	CI	n/N	%	CI	n/N	%	CI
Severe and moderate visual deficiency <3.2/10	8	1.30	0.38-3.17	4	0.21	0.03-0.73	1	0.50	0.01-4.51
Mild visual deficiency <5/10	58	10.26	7.18-14.10	70	3.59	2.55-4.91	7	5.51	1.44-13.87
No visual deficiency									
5/10-6.3/10	297	41.25	36.16-46.48	562	31.00	28.15-33.98	44	39.88	27.20-53.64
8/10	150	24.60	20.14-29.51	416	23.11	20.52-25.85	21	29.34	17.38-43.81
10/10	164	22.58	18.46-27.14	739	42.09	38.99-45.23	29	24.78	14.64-37.45

CI: Confidence Interval

* Observed data, denominators vary according to the number of missing data for each variable. Percentages are weighted to took into account the differences in survey design between gestational age groups.
